# The Role of Auxin in the Pattern Formation of the Asteraceae Flower Head (Capitulum)^1^

**DOI:** 10.1104/pp.18.01119

**Published:** 2018-11-20

**Authors:** Nicholas Zoulias, Sascha H. C. Duttke, Helena Garcês, Victoria Spencer, Minsung Kim

**Affiliations:** Faculty of Biology, Medicine and Health, University of Manchester, Oxford Road, Manchester M13 9PT UK

## Abstract

Nature often creates complex structures by rearranging pre-existing units. One such example is the flower head (capitulum) in daisies, where a group of flowers (florets) and phyllaries (modified bracts) are arranged to superficially mimic a single flower. The capitulum is a key taxonomical innovation that defines the daisy family (Asteraceae), the largest flowering plant group. However, patterning mechanisms underlying its structure remain elusive. Here, we show that auxin, a plant hormone, provides a developmental patterning cue for the capitulum. During capitulum development, a temporal auxin gradient occurs, regulating the successive and centripetal formation of distinct florets and phyllaries. Disruption of the endogenous auxin gradient led to homeotic conversions of florets and phyllaries in the capitulum. Furthermore, auxin regulates floral meristem identity genes, such as *Matricaria inodora RAY2* and *M*. *inodora LEAFY*, which determine floret and phyllary identity. This study reveals the mechanism of capitulum patterning and highlights how common developmental tools, such as hormone gradients, have independently evolved in plants and animals.

## Abstract (precis)

Auxin and key flower meristem genes play a pivotal role in patterning of the capitulum, a key innovation that taxonomically defines the daisy family.

A pseudanthium (“false flower”) is one of the most successful traits that has recurred throughout the evolution of angiosperms (Harris, 1999). In a pseudanthium, a group of flowers and bracts (modified leaves) have evolved to mimic a single flower. The most common pseudanthium is the capitulum of the Asteraceae (daisy, sunflower) family. A typical capitulum consists of many flowers (florets) and phyllaries (modified bracts) compressed into a single structure (Fig. 1, A–C, shown in *Matricaria inodora*, also known as *Tripleurospermum inodorum*, or “scentless chamomile”). Capitula commonly have two types of florets: ray and disc florets. Ray florets have bilateral floral symmetry with three fused ventral petals protruding like a tongue shape, whereas disc florets have radial symmetry with five evenly sized petals. Disc florets are usually perfect flowers, although some ray florets are pistillated in some species (Weberling, 1989). Adoption of this characteristic capitulum is proposed to be the key to the evolutionary success of Asteraceae as one of the largest plant families (Cronquist, 1981). In most naturally occurring cases, pattern formation of the capitulum is precisely controlled, with phyllaries, ray florets, and disc florets positioned in a centripetal order in the capitulum, which mimic sepals, petals, and anthers, respectively (Fig. 1, B and C). The formation of phyllaries and florets is asynchronous: commonly acropetally (forming from the margin to center of the capitulum), but in some species bidirectionally (Harris, 1995).

**Figure 1.**
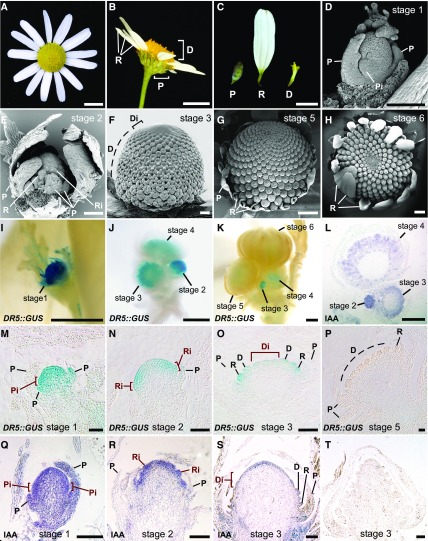
Capitulum morphology and development. A–C, Nontreated *M*. *inodora* capitula (A and B), and phyllaries and florets (C). D–H, SEM images of developing nontreated *M*. *inodora* capitula. I to K, GUS expression in *DR5*::*GUS S*. *vulgaris* capitula. A young capitulum (I, stage 1). Clusters of capitula showing different developmental stages (J and K, stages 2 to 6). M–P, Sections of *DR5*::*GUS S*. *vulgaris* capitula at different stages of development. GUS concentration decreases as the capitulum develops in *DR5*::*GUS* lines. L and Q–S, IAA immunolocalization in developing *S*. *vulgaris* (L) and *M*. *inodora* (Q–S) capitula. T, Negative control (no primary antibody). Scale bars = 5 mm (A and B), 2 mm (C), 100 μm (D–F, M–T), and 500 µm (G and H, I–L). P, phyllary; Pi, incipient phyllary primordium; R, ray floret; Ri, incipient ray floret primordium; D, disc floret; Di, incipient disc floret primordia.

Although little is known about the patterning mechanism(s), floret identity (ray floret vs. disc floret) appears to be controlled by the flower symmetry gene *CYCLOIDEA* (*CYC*). *CYC* is a member of the *TCP* (*TEOSINTE BRANCHED1* in maize [*Zea mays*], *CYC* in snapdragon [*Antirrhinum majus*], and *PROLIFERATING CELL FACTORS1* and *2* in the rice [*Oryza sativa*]) gene family, and *Antirrhinum cyc* mutants showed a flower symmetry change from bilateral to radial (Luo et al., 1996). *CYC* homologs have been independently recruited during acquisition of bilateral flower symmetry across angiosperms (Busch and Zachgo, 2007; Zhang et al., 2010; Howarth et al., 2011; Hileman, 2014; Zhong and Kellogg, 2015; Spencer and Kim, 2018). Many *CYC* homologs are expressed in developing ray florets and determine ray floret identity in several Asteraceae species including common groundsel (*Senecio vulgaris*), gerbera (*Gerbera hybrida*), and sunflower (*Helianthus annuus*; Broholm et al., 2008; Kim et al., 2008; Chapman et al., 2012; Juntheikki-Palovaara et al., 2014; Fambrini et al., 2018). In *S*. *vulgaris*, presence/absence of ray florets in the capitulum is controlled by two *CYC* genes, *RAY1* and *RAY2*, and overexpression of *RAY2* led to the formation of extra ray florets or tubular rayflorets (Kim et al., 2008). Consistently, overexpression of *H*. *annuus CYC2c*, converted disc florets to ray florets, generating capitula with only ray florets in sunflower (Chapman et al., 2012). In gerbera, several ABC genes (*MADS*-box genes) were differentially expressed between ray and disc florets, suggesting that these genes are also involved in determining floret identity (Laitinen et al., 2006). Recently, it has also been reported that *LEAFY* (*LFY*) plays a role in capitulum development. *LFY* is a key regulator of floral meristem identity, and *lfy* mutants reportedly make secondary inflorescences with cauline leaves instead of flowers in Arabidopsis (*Arabidopsis thaliana*) and *Antirrhinum* (Coen et al., 1990; Weigel et al., 1992). In gerbera, a *LFY* homolog, *GhLFY*, was expressed in the center of the young capitulum where florets are formed, and severe *GhLFY* RNAi plants generated a capitulum with only phyllaries (Zhao et al., 2016).

It has been shown that the auxin pathway interacts with the *LFY* pathway, suggesting a possible role for auxin in controlling capitulum patterning. In Arabidopsis, auxin accumulation preceded *LFY* expression (Li et al., 2013), and application of auxin onto inflorescences up-regulated *LFY* mRNA and protein (Yamaguchi et al., 2013). Auxin has been previously suggested to play a morphogen-like or a morphogenic trigger role in plant development, which is still open to debate (Bhalerao and Bennett, 2003; Benková et al., 2009; Möller and Weijers, 2009; Lau et al., 2011; Finet and Jaillais, 2012). An auxin gradient was reported in several plant tissues such as the secondary vasculature, the female gamete, and the root tip (Uggla et al., 1996; Sabatini et al., 1999; Friml et al., 2003; Schrader et al., 2003; Pagnussat et al., 2009; Brunoud et al., 2012; Dubreuil et al., 2018). This suggests that auxin can provide positional cues for tissue specification in a concentration-dependent manner. Although studies on *CYC* genes and *GhLFY* suggest their roles in specifying different types of florets or floret identity over phyllary, it is still not clear how patterning of florets and phyllaries in a capitulum is established, and whether auxin is involved in this process.

## RESULTS AND DISCUSSION

### Asynchronous Formation of Phyllaries and Florets in a *M*. *inodora* Capitulum

A *M*. *inodora* capitulum consists of green phyllaries, white ray florets, and yellow disc florets (Fig. 1, A–C). These three structures are asynchronously formed in the developing capitulum. The *M*. *inodora* capitulum meristem successively generates phyllaries (Fig. 1D, stage 1), ray (Fig. 1E, stage 2), and disc florets (Fig. 1F, stages 3 and 4), which is followed by rapid petal elongation of the ray florets during stages 5 and 6 (Fig. 1, G and H). A developing capitulum (stages 1–3) consists of a pool of fast-dividing undifferentiated cells in the center of the meristem dome with phyllaries and florets forming in the peripheral zone. As the capitulum develops to form phyllaries and florets, undifferentiated cell daughters are continuously recruited from the central dome to give rise to a spiral of incipient phyllary and floret primordia where phyllary or floret identity is determined. Initially, the capitulum forms the spiral of incipient phyllary primordia (Fig. 1D, P_i_, stage-1 capitulum), followed by the spiral of incipient ray (Fig. 1E, R_i_, stage 2), and disc (Fig. 1F, D_i_, stage 3) floret primordia consecutively.

### A Temporal Auxin Gradient Is Established during Capitulum Development

To determine whether auxin provides a developmental cue for capitulum pattern formation, we first investigated the presence of an innate auxin accumulation in the developing capitula. A visual auxin reporter line, *DR5*::*GUS* (β-glucuronidase; Ulmasov et al., 1997a), was generated in a transformable Asteraceae model species, *S*. *vulgaris* (Kim et al., 2008). Out of six independent lines, five lines showed a similar β-glucuronidase (GUS) expression pattern in developing capitula (Fig. 1, I–K) as well as in other expected tissues such as root tips (Supplemental Fig. S1B), young leaves, and vasculature. Notably, different stages of capitulum development showed different levels of GUS expression. In the capitula, GUS expression levels were high (dark blue) in stages 1 and 2 (Fig. 1, I and J) but low (pale blue) in stages 3 and 4 (Fig. 1, J and K), followed by a further decrease in stages 5 and 6 (Fig. 1K). Moreover, quantification of GUS activity (via Fluorescent β-Galactosidase Assay [MUG]) showed that GUS activity decreased significantly as the capitulum developed (Supplemental Fig. S1A). More importantly, GUS expression differed depending on the type of incipient primordia (Fig. 1, M–O); GUS expression was the highest in the incipient phyllary primordia (P_i_; Fig. 1M), lower in the incipient ray floret primordia (R_i_; Fig. 1N) and the lowest in the incipient disc floret primordia (D_i_; Fig. 1O). To determine whether the GUS activity in *DR5*::*GUS* plants faithfully reflected auxin accumulation in *S*. *vulgaris* capitula, we also visualized auxin accumulation by immunolocalization using an anti-indole-3-acetic acid (IAA) antibody. IAA immunolocalization provided an additional line of evidence that auxin concentration decreased as the capitulum developed (Fig. 1L). Consistent with *DR5*::*GUS* lines, auxin concentrations also differed among different incipient primordia in *S*. *vulgaris* (Fig. 1L).

To further investigate whether this auxin distribution pattern in a capitulum is conserved in different Asteraceae species, we performed IAA immunolocalization in *M*. *inodora*. In *M*. *inodora*, IAA immunolocalization also showed a similar auxin distribution pattern; auxin was the highest in the incipient phyllary primordia (P_i_; Fig. 1Q), lower in the incipient ray floret primordia (R_i_; Fig. 1R), and the lowest in the incipient disc floret primordia (D_i_; Fig. 1S, and negative control in Fig. 1T). Furthermore, immunolocalization showed that PIN-FORMED1 (PIN1), an auxin efflux carrier (Gälweiler et al., 1998), and YUCCA1, a key enzyme for auxin biosynthesis (Zhao et al., 2001), were present in the developing capitula, suggesting their active roles in establishing the auxin distribution in the *M*. *inodora* capitulum (Supplemental Fig. S2, A–H). Taken together, our results showed that a temporal auxin gradient occurs in the developing capitula. As the capitulum sequentially forms phyllaries,ray and disc florets, the auxin concentration decreases in the respective incipient primordia where phyllaries, ray and disc florets are being generated. This suggests an intriguing hypothesis that auxin may play a critical role in determining the identity of these lateral organs; a high auxin concentration in the incipient primordia is likely to generate phyllaries, while low and lower auxin concentrations may generate ray and disc florets, respectively.

### Disruption of Auxin Accumulation Led To Homeotic Conversions of Phyllaries and Florets in the Capitulum

**Figure 2.**
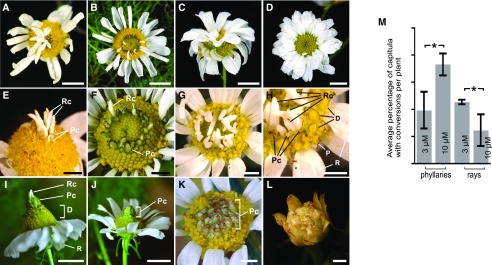
Auxin application induced homeotic conversions in the capitulum. Phenotypes of capitula sprayed with 3-μM (A–I) and 10-μM (J–L) IAA, showing conversion of disc florets into both ray florets and phyllaries (A–I) or solely into phyllaries (J–L). A–D, Capitula with fully developed converted phyllaries and ray florets. E–H, Initial developing stages of converted phyllaries and ray florets after IAA treatments. H, Close-up of (G) showing the order of converted phyllaries and ray florets. Scale bars = 5 mm (A–D, I–J), 2 mm (E–H, K and L). M, Quantification of phyllary and ray floret conversion after IAA treatments. Each error bar represents the mean ± SE. Values marked by asterisk are significantly different (*P* = 0.04 for ray florets and *P* = 0.04 for phyllaries; two-tailed *t*-test analysis). Pc, converted phyllary; Rc, converted ray floret.

To test whether different auxin concentrations determine floret and phyllary identity in a capitulum, we manipulated the endogenous auxin distribution in *M*. *inodora* by applying IAA, a naturally occurring auxin. We applied a range of IAA concentrations (1 μM, 3 μM, 10 μM and 50 μM) onto young capitula (approximately stage 3). Results showed that whereas 1 μM had no effect and 50-μM IAA damaged the whole plants, 3-μM and 10-μM concentrations caused the conversion of disc florets into either phyllaries or ray florets (Fig. 2). We sprayed 1,815 capitula with 3-μM– or 10-μM–IAA concentrations and 422 capitula showed conversion of disc florets into either phyllaries or ray florets (Supplemental Tables S1 and S2). No conversion was observed in any of the 115 mock-sprayed capitula (Supplemental Table S1). Notably, the converted phyllaries and ray florets showed normal wild-type morphology and color, suggesting that these conversions are homeotic. The position of the converted ray florets or phyllaries was variable; converted ray florets and phyllaries were formed in the center (Fig. 2A), in the middle (Fig. 2, B and C), or in the margin (next to the innate ray florets, Fig. 2D) of the capitulum dome. These different positions of converted ray florets and phyllaries reflected the location of the primordia forming region when the auxin was applied. The primordium-forming region is close to the margin of the capitulum at early stage 3, but moves to the center of the capitulum later in stage 3. In later stages (stages 4–6), capitula did not exhibit notable conversion phenotypes when sprayed with exogenous auxin, indicating a limited developmental window of time and potency for reprogramming in the incipient primordia. In addition, local applications of 10-μM–IAA concentrations at one side of the stage-2 capitulum periphery could induce the conversion of disc florets to ray florets at the site of IAA application (Supplemental Fig. S1C, arrows; and Supplemental Table S3). Together, these results indicate that exogenous auxin application was able to influence the developmental process that determined phyllary and floret identities in the region of incipient disc floret primordia. Notably, converted phyllaries and ray florets in IAA-treated capitula were always formed sequentially in the order of phyllaries, ray florets, and disc florets from the periphery to the center of the capitulum (Fig. 2, E–I), mirroring the naturally occurring pattern of a nontreated capitulum (Fig. 1B). We believe this is a clear indication that auxin concentration plays a key role in determining the identity of these organs. It is probable that the perceived auxin in the cells, newly formed after exogenous IAA application, is lower than directly treated cells, indicating that the initial higher auxin concentration induces phyllaries, followed by the formation of ray and disc florets in response to declining auxin levels. In fact, among 884 capitula sprayed with 3-μM IAA, 79 capitula showed disc floret–phyllary conversion and 98 showed disc floret–ray floret floret conversion, whereas among 931 capitula sprayed with 10-μM IAA, 154 had disc floret–phyllary conversion and 57 had disc floret–ray floret conversion (Supplemental Table S1). Capitula treated with 3-μM IAA showed a significantly (*P* = 0.04) higher rate of conversion to ray florets, whereas capitula treated with 10-μM IAA showed a significantly (*P* = 0.04) higher rate of phyllary conversion (Fig. 2M; Supplemental Table S2). These experiments showed that different auxin concentrations appear to correlate to the identity of phyllary and florets. It also supports the hypothesis that the innate temporal auxin gradient (Fig. 1, M–S) in the incipient primordia regulates lateral organ identity in native capitulum development.

### Auxin Regulates Floret Identity Genes Such As *MiRAY2* and *MiLFY*

**Figure 3.**
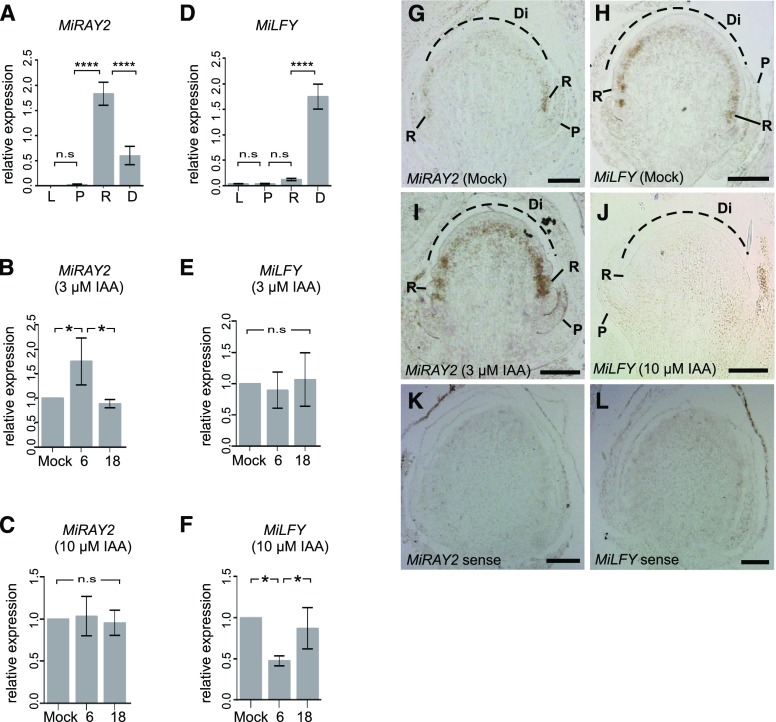
Auxin regulates floret identity genes in *M*. *inodora* capitula. A–F, RT-qPCR of *MiRAY2* (A–C) and *MiLFY* (D–F) on untreated dissected leaves, phyllarys, and florets (A and D) and on stage-3 whole capitula treated with 3-μM (B and E) and 10-μM (C and F) IAA. The *X* axes in (A) and (D) represent dissected organs and in (B), (C), (E), and (F), “6” and “18” represent hours after auxin application. The *Y* axes indicate the relative expression of *MiRAY2* and *MiLFY*. Each bar represents the mean ± SE. Values marked by asterisk are significantly different (*P < 0.05, ^****^P < 0.0001), with “n.s.” as non-significant, at *P* > 0.05 (one-way analysis of variance with post-hoc Tukey’s multiple comparison test). G to L, *M*. *inodora* in situ hybridizations using *MiRAY2* (G, I, and K) and *MiLFY* (H, J, and L) probes in mock-treated (G and H) and 3-μM (I) or 10-μM (J) IAA-treated stage-3 capitula. K and L, Sense probe control. Scale bars = 50 μm. L, leaves.

To explore how the temporal auxin gradient could be translated into mechanisms that modulate phyllary and floret identities, we investigated the effect of auxin on known floret meristem identity genes, *RAY2* and *LFY*. In *S*. *vulgaris*, exogenous auxin (3-μM IAA) application induced ray floret conversion, which phenocopied capitula overexpressing *RAY2* (Kim et al., 2008), implying a positive regulatory relationship between auxin and *RAY2* (Supplemental Fig. S1, F and G). This observation is consistent with other plant species, in which auxin also regulates *TCP* genes (Das Gupta et al., 2014). To determine whether the expression of the *M*. *inodora RAY2* ortholog (*MiRAY2*) was similar to *S*. *vulgaris RAY2*, we cloned (orthology was confirmed by phylogenetic analyses, see Supplemental Fig. S1I) and determined the expression of *MiRAY2* in young untreated *M*. *inodora* developing phyllaries and florets from stage 3 to stage 6 capitula. Reverse transcription quantitative PCR (RT-qPCR) results showed that consistent with *S*. *vulgaris RAY2* (Kim et al., 2008), *MiRAY2* was strongly expressed in ray florets (Fig. 3A). To further investigate whether auxin regulated ray floret conversions via *MiRAY2* activity, we determined the expression of *MiRAY2* on stage-3 capitula after IAA treatment. RT-qPCR results showed that auxin affected the expression levels of *MiRAY2* in a concentration-dependent manner. The expression level of *MiRAY2* was up-regulated in *M*. *inodora* capitula 6 h after 3-μM–IAA treatment (Fig. 3B), whereas 10-μM–IAA treatment had no significant effect (Fig. 3C). Moreover, RNA localization by in situ hybridization showed that in untreated stage-3 capitula, *MiRAY2* was expressed only in the ray florets (Fig. 3G), whereas in IAA-treated capitulum, *MiRAY2* was expressed in the center of the capitulum dome as well as in ray florets (Fig. 3I). These results suggest that auxin regulates *MiRAY2* expression in *M*. *inodora*, and perhaps the formation of converted ray florets in auxin-treated capitula were through up-regulation of *MiRAY2* expression.

As phyllaries resemble cauline leaves and down-regulation of *LFY* converted ray and disc florets into phyllaries in *S*. *vulgaris* transgenic plants (Supplemental Fig. S1H) and gerbera (Zhao et al., 2016), we hypothesized that auxin regulates phyllary conversions via *MiLFY* activity. We therefore cloned *MiLFY* and determined its expression patterns in *M*. *inodora*. RT-qPCR analysis showed that the expression of *MiLFY* was low in young developing phyllaries, compared to ray or disc florets in untreated *M*. *inodora* capitula (Fig. 3D). Although both ray and disc florets showed higher levels of *MiLFY* expression than that of phyllaries, only disc florets were statistically higher in this RT-qPCR result. However, our in situ hybridization (Fig. 3H) and anti-LFY immunolocalization data (Supplemental Fig. S2, I–K) clearly showed that young ray floret primordia had *MiLFY* expression as strong as young disc floret primordia. Immunolocalization using an anti-LFY antibody showed that initially LFY was low in phyllary primordia, then later up-regulated in both ray and disc floret primordia (Supplemental Fig. S2, I–K). Our RT-qPCR results showed that the expression levels of *MiLFY* in the capitula (stage 3) were down-regulated 6 h after the 10-μM–IAA treatment (Fig. 3F), whereas 3-μM–IAA treatment had no effect (Fig. 3E). Furthermore, in situ hybridization results confirmed that *MiLFY* was expressed in ray and disc floret primordia (Fig. 3H), and was down-regulated in capitulum (stage 3) after 10-μM–IAA treatment (Fig. 3J). Taken together, the expression analyses (Fig. 3, B and F) combined with the phenotypic data (Fig. 2; Supplemental Table S1) demonstrate that it is likely that auxin regulates flower meristem genes such as *MiRAY2* and *MiLFY* in a concentration-dependent manner, which in turn determines phyllary and floret identities in *M*. *inodora* capitulum.

### A Model for Capitulum Patterning

**Figure 4.**
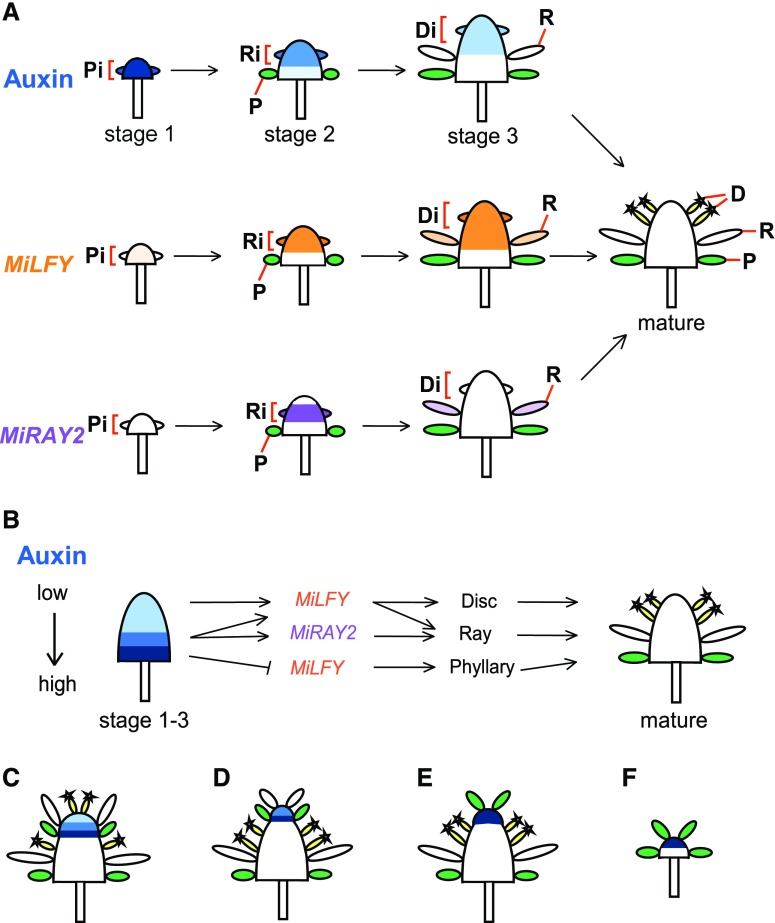
Schematic models of how auxin gradients determine capitulum patterning. A, Wild-type *M*. *inodora* capitula from young to mature stages and their respective auxin concentrations (in shades of blue) as well as the expressions of *MiLFY* (in orange) and *MiRAY2* (in purple). Brackets represent the meristematic regions where phyllaries, ray florets, and disc florets will be initiated (Pi, Ri, and Di). B, Capitulum showing superimposed auxin gradients of different developmental stages and downstream gene regulation. C–F, Schematic models representing the homeotic conversion phenotypes shown in Fig. 2 and the respective auxin gradients. Exogenous auxin application to stage-3 capitulum induces an ectopic auxin gradient in the center of the capitulum, which promotes the reappearance of phyllaries and ray florets (C–E). Moreover, a range of auxin levels within the ectopic gradient will determine the final capitulum morphology. An ectopic gradient consisting of all the auxin levels represented in stages 1, 2, and 3 (A) will generate phyllaries, ray florets, and disc florets sequentially in the center of the capitulum (C; and see Fig. 2, B and C). An ectopic gradient consisting of the auxin levels of stages 1 and 2 will generate phyllaries and ray florets in the center of the capitulum (D; and see Fig. 2, A, E, and I), whereas a gradient consisting of stage-1 auxin levels will generate only phyllaries (E and F; and see Fig. 2, J–L).

Our results consistently suggest that an endogenous auxin gradient provides a developmental cue for capitulum patterning in a concentration-dependent manner (see models in Fig. 4A). Auxin concentration changes from high (dark blue) to low (pale blue) in the region of the incipient primordia (Fig. 4A, P_i_, R_i_, D_i_, in brackets) as the capitulum forms phyllaries, ray florets, and disc florets consecutively. Once the primordia emerge and expand, auxin concentration decreases (pale blue to white) in the region where phyllaries (stage 2), ray (stage 3), and disc (mature stage) florets are already formed. *MiLFY* (in orange) expression is very low in the stage-1 capitulum but is later strongly expressed in the incipient ray and disc floret primordia (Fig. 4A, stages 2 and 3), whereas *MiRAY2* (in purple) is expressed in the incipient ray floret primordia (Fig. 4A, stage 2). Initially high auxin levels (Fig. 4B, stage-1 capitulum) repress *MiLFY* expression (or activate/maintain low *MiLFY* expression) to form phyllaries. As the capitulum develops further (stages 2–3), auxin levels decrease, and consequently *MiLFY* (in orange) is turned on in the incipient ray and disc floret primordia (R_i_ and D_i_, respectively), allowing ray and disc florets to form. Simultaneously, auxin also declines to a certain level (stage 2) to up-regulate *MiRAY2* expression (in purple) in the region where ray florets will be formed (*R_i_*). Finally, in the capitulum at stage 3, auxin and *MiRAY2* expression levels decrease further in the incipient disc floret primordia (D_i_), which in turn results in disc floret formation (Fig. 4B).

According to this model, various homeotic conversion phenotypes shown in Figure 2 can be explained with the respective auxin gradients. Exogenous auxin application to a stage-3 capitulum induces an “ectopic” auxin gradient in the center of the capitulum, which promotes the reappearance of phyllaries and ray florets (Fig. 4, C–E). Moreover, a range of auxin levels within the ectopic gradient will determine the final capitulum morphology. In this model, an ectopic gradient consisting of all the auxin levels represented in stages 1, 2, and 3 will generate phyllaries, ray, and disc florets sequentially in the center of the capitulum (Fig. 4C). An ectopic gradient consisting of the auxin levels of stages 1 and 2 will generate phyllaries and ray florets in the center of the capitulum (Fig. 4D), whereas a gradient consisting of only stage-1 auxin levels will generate only phyllaries (Fig. 4, E and F).

Furthermore, our results support a morphogen-like role for auxin in capitulum development. Although the classical concept of spatial morphogen gradients is based on the “French flag” model (Wolpert, 1969), recent evidence suggests an equal importance of temporal and spatial distribution of morphogen gradients in many developmental processes (Jaeger et al., 2008). In our model, the dynamic nature of the temporal auxin gradient is essential for capitulum patterning where distinct phyllaries and florets are “asynchronously” generated. Although previous studies have shown that auxin determines lateral organ position (Sabatini et al., 1999; Reinhardt et al., 2000, 2003; Friml et al., 2003; Koenig et al., 2009), and cell and tissue patterning (Uggla et al., 1996; Schrader et al., 2003; Pagnussat et al., 2009; Sorefan et al., 2009), here we provide insight into auxin’s role in determining phyllary (leaf) and floret (flower) identities during plant development. Our results showed that auxin regulates downstream flower meristem genes such as *MiRAY2* and *MiLFY*. Further investigation into how a broad range of auxin concentrations affects the expression of these genes will be informative. It will also be interesting to see how auxin gradients act on these genes in different species, as auxin may control *LFY* expression differently in Arabidopsis and *M*. *inodora*; 10-μM 2,4-D activated *LFY* in Arabidopsis (Yamaguchi et al., 2013), but 10-μM–IAA treatment inhibited *MiLFY* in *M*. *inodora*.

Previously, it has been proposed that surface tension and mechanical buckling of the capitulum dome is also important for capitulum patterning (Hernandez and Green, 1993); application of physical compression to the young sunflower capitulum could alter the capitulum patterning with ectopic bracts visible in the center (Hernandez and Green, 1993). Therefore, it is also possible that biophysical components may be involved in generating the phenotypes seen in our IAA-sprayed capitula. However, our scanning electron microscope (SEM) images did not show obvious buckling or growth behavioral changes in IAA-treated capitula (Supplemental Fig. S1D). Alteration of capitulum pattern by surface tension and mechanical buckling may be through redirecting auxin trafficking, as it has been shown that biomechanical tension can relocate auxin efflux carrier PIN proteins and thus the auxin flow in the shoot apical meristem (Kierzkowski et al., 2012; Nakayama et al., 2012). As auxin also has a role in cell wall loosening (Rayle and Cleland, 1992), it is also plausible that mechanical buckling is a consequence of the excessive auxin accumulation in the capitulum. In sunflower, cylindrical wounding in the center of the capitulum induced phyllaries, ray florets, and disc florets, centripetally (Hernandez and Palmer, 1988), as was seen in our IAA-sprayed capitulum. Possibly an ectopic auxin gradient may be involved in generating wound-induced phyllaries, ray florets, and disc florets. An ectopic centripetal auxin gradient can be formed as the wound physically blocks the auxin flow, leading to excessive auxin accumulation in the rim of the wound.

Together, this new role of auxin further sheds light on how animals and plants have evolved comparable principles in their developmental processes, including similar components such as gradient cues and morphogen-regulated target genes that determine lateral organ pattern formation. Our results uncover the mechanism controlling pattern formation in the capitulum, the most common pseudanthium seen in nature (Cronquist, 1981). This highlights how convergent evolution can invent a complex structure such as a capitulum by introducing new developmental machinery (an auxin gradient) to control pre-existing target genes such as *RAY2* and *LFY* that have conserved functions in solitary flowers.

## MATERIALS AND METHODS

### Plant Materials

All plant materials were grown in a growth chamber under long-day conditions (150 μmol m^−2^ S^−1^, 16-h light, 24°C day temperature) in 4-inch square pots, on Sinclair compost until flowering. Photos of capitula were taken using a D3100 camera with a 105-mm Nikkor macro lens (both by Nikon). Plants for tissue culture were grown from seeds, in Magenta boxes. Seeds were sterilized with 20% (v/v) sodium hypochlorite (domestic grade) for 10 min, then washed three times for 15 min with sterile water before transfer to seed germination media (half-strength Murashige & Skoog [MS, w/v] media, 0.8% [w/v] plant agar [Melford] at pH 5.8) in petri dishes. Seeds then were treated with 0.1% Gibberellic Acid A3 (Melford) to increase the rate of germination. Plates were then transferred to a Percival tissue culture cabinet (22°C, 16 h light, 100 μmol m^−2^ S^−1^). After one week, seedlings were transferred to Magenta boxes containing the culture media (full-strength MS [w/v] media, 3% Suc [w/v], 0.8% [w/v] plant agar [Melford] at pH 5.8) and left to grow for one month before being used as leaf explants for tissue culture transformation.

### Auxin Treatments

The stages 2 to 3–developing capitula were sprayed with an aqueous solution containing 1% Methanol, 0.5% TWEEN 20, and 1% dimethyl sulfoxide (DMSO; for mock); or containing various auxin concentrations, 1-μM, 3-μM, 10-μM, or 50-μM IAA. Once sprayed, plants were then covered and left overnight (for 16 h). First phenotypic capitula were observed two to four weeks after treatments and phenotypic capitula continued to be observed for up to two months. Local treatment experiments were performed by applying lanolin wax mixed with 10-μM IAA or DMSO (mock) on stage-2 *Matricaria inodora* capitula, with phyllaries dissected away to expose the developing capitulum meristem as described in Reinhardt et al. (2000).

### Plant Transformation

*Senecio vulgaris* transformation was performed using *Agrobacterium tumefaciens* (GV3101) with pBI121 containing the DR5 promoter and the *NEOMYCIN PHO​SPH​OTR​ANS​FER​ASE​II* gene. Leaves were harvested from one-month-old plants (Magenta grown) and cut into roughly 2 cm^2^ explants for transformation. Explants were incubated for 20 min with an *Agrobacterium* solution (GV3101 was resuspended to an optical density of 0.6 to 0.8 [600 nm] in a 3% [w/v] Suc solution containing full-strength MS salts and 100 μm acetosyringone) at room temperature (RT) in the dark. After incubation, the explants were dried on filter paper to remove excess *Agrobacterium* and incubated on coculture media (full-strength MS salts, 3% Suc [w/v], thidiazuron [1 mg/L], and naphthaleneacetic acid [0.1 mg/L] pH to 5.8 with NaOH) for 3 d 22°C in the dark. After the dark incubation, explants were transferred to fresh coculture plates containing antibiotics (Kanamycin 40 mg/L and Cefotaxamine 250 mg/L) and left for two weeks in a Percival tissue culture cabinet (22°C, 16 h light and 100 μmol m^−2^ S^−1^). Explants were continuously transferred onto fresh coculture media every two weeks until the appearance of calli with shoots. Once shoots were formed, they were removed from calli and transferred to Magenta boxes containing root induction media (full-strength MS salts and 3% [w/v] Suc, pH 5.8, with antibiotics Kanamycin 100 mg/L and Cefotaxamine 250 mg/L). Shoots with visible roots were transferred to fresh media in Magenta every month or sooner if needed. Once transgenic plants had a developed root system, they were transferred to soil and covered with a humidity lid for ∼5 d. The cover was slowly removed over the period of a week. The whole process from explant to transgenic plant took from six to 10 months. The presence of the transgene was confirmed using PCR and visual markers (GUS staining) if applicable.

### Constructs

All PCRs were performed using Phire Hot Start DNA Polymerase (Thermo Fisher Scientific), dNTPs from Bioline, and primers synthesized by Eurofins. The *DR5*::*GUS* construct was made by PCR amplification of the DR5 promoter from the pUC19 plasmid containing DR5 sequences (Ulmasov et al., 1997b), with primers that contained *Cla*I and *Xba*I sites. After digestions, the DR5 fragment was ligated into pBI121 (Clontech).

### GUS Staining

Transgenic plants transformed with the βGUS reporter system were visualized using established methods (Sessions et al., 1998). In brief, transgenic and wild-type capitula of different developmental stages were collected and fixed in 90% (v/v) ice cold acetone for 20 min. Capitula were then washed for 10 min in GUS staining buffer (100 mM potassium phosphate buffer pH 7.0, 10 mM [ethylenediaminetetraacetic acid, EDTA], 0.1% Triton X-100, 0.5 mM potassium ferricyanide, 0.5 mm potassium ferrocyanide). Fresh GUS staining buffer containing the GUS substrate (5-Bromo-4-chloro-3-indolyl-β-d-GlcA, cyclohexyl ammonium salt [2 mM]) was then applied to the capitula and was vacuum-infiltrated for 10 min. Tissue was then incubated in the dark at 37°C overnight for a maximum of 24 h (average time was 20 h). The next day, capitula were dehydrated with 30% ethanol then fixed with an acidic formaldehyde solution (3.7% formaldehyde, 50% ethanol, 5% acetic acid) for 30 min. Tissue dehydration continued with 70%, 85%, 90% and 100% ethanol (30 min each step). The stained capitula were then photographed using a Leica MZ6 stereomicroscope with a Nikon D3100 camera attached. Stained capitula were infiltrated with a solution of Histo-Clear II (Scientific Laboratory Supplies)/100% ethanol (1:1) and Histo-Clear II for 30 min each. Capitula were then added to 100% liquid paraffin (Sigma-Aldrich) at 58°C overnight before being embedded into paraffin blocks and sectioned (14-μm thickness). Sectioned material had the paraffin removed with 2 × 10-min washes in Histo-Clear II before having coverslips mounted with Roti-Histo Kit II permount (Roth). Sectioned material was imaged on a Leica DMR fitted with a SPOT Insight 4.0-Mp Color F-Mount (SPOT Imaging Solutions) using SPOT advanced software (SPOT Imaging Solutions). Phase contrast and Nomarski Interference Contrast settings were used for some pictures.

### RT-qPCR

For RT-qPCR analysis of individual phyllaries and florets, total RNA was extracted from comparable young developing pyllaries, ray florets, and disc florets (sized 0.5 mm to 1 mm) that were dissected from stage 3 to stage 6 *M*. *inodora* capitula under a dissecting microscope (Nikon). For the analysis of auxin-sprayed capitula, capitula were treated as previously described in the "Auxin Treatments" section and collected at 0 h, 6 h, and 18 h after auxin application. Total RNA was extracted using the RNeasy Plant Mini Kit (Qiagen). After DNase I (Promega) treatment, cDNAs were synthesized using Superscript II (Invitrogen) according to the manufacturer’s description. Primers for qPCR were designed using Primer3 software (Untergasser et al., 2007) on previously cloned sequences of target genes as well as sequences obtained from the National Center for Biotechnology Information (primer sequences in Supplemental Table S4). Annealing temperatures were kept to ±1°C of 60°C with a target GC content of 50% to 60%. RT-qPCR was performed on an ABI PRISM 7000 using a SensiFAST SYBR Hi-ROX Kit (Bioline). Reactions were performed according to the manufacturer’s specifications with final concentrations of 500 nM for forward and reverse primers, and 10 ng of RNA per reaction. All samples were run as biological triplicates with technical quadruplicates. A melting curve analysis was performed for each run to ensure only single products were made. Samples were normalized to *RIBOSOMAL PROTEIN SUBUNIT9* and 18s rRNA, and their expression determined using the comparative threshold cycle method (Schmittgen and Livak, 2008). The PCR efficiency of each target gene was calculated using LinReg (Hårdstedt et al., 2005).

### In Situ Hybridizations

In situ hybridizations were performed on wild-type and auxin-treated stages 2 to 3–developing capitula of *M*. *inodora*. In situ hybridizations of *MiLFY* and *MiRAY2* were performed using the protocol described by Coen et al. (1990). In brief, young capitula (stages 2 to 3) were auxin-treated, fixed in 4% (v/v) paraformaldehyde (PFA) solution 6 h after treatment, and embedded and sectioned as described in Coen et al. (1990). Sectioned tissue was deparaffinized with 2× 10 min treatments with Histo-Clear II. Tissues were rehydrated in a decreasing ethanol series (100% [v/v], 95% [v/v], 90% [v/v], 80% [v/v], 60% [v/v], 30% [v/v], H_2_O). Slides were then treated for 25 min at 37°C with 0.065 mg/mL of proteinase K (Sigma-Aldrich). Proteinase K digestion was stopped with a 0.2% (v/v) solution of Gly (Sigma-Aldrich) before the samples were fixed in 4% PFA. Fixed sections were acetylated with acetic anhydride then dehydrated back through the ethanol series and left at 4°C until probe hybridization. Fragments of *MiLFY* and *MiRAY2* genes were ligated into pDRIVE (Qiagen) and pGEM-T easy (Promega), respectively. M13 forward and reverse primers were used to amplify both the T7 and SP6 promoters contained on the plasmids as well as the gene fragment. Sense and anti-sense single-stranded RNA probes were transcribed with the Digoxigenin labeling mix (Roche Applied Science), using T7 and SP6 RNA polymerases according to the manufacturer’s specifications. Probes were prepared for hybridization by being heated to 100°C with 50% formamide (2 μL to 5 μL of probe with formamide up to a final volume of 20 μL), then placed on ice until needed. Hybridization solution (40% formamide, 1× Denhardt’s reagent, 9 × 10^−5^ mg/mL tRNA, 10% dextran sulfate, and 1× in situ salt solution [0.3 M NaCl, 10 mM Tris-HCl, 5 mM EDTA, 5 mM Na_2_HPO_4_, and NaH_2_PO_4_2H_2_O]) at 85°C was then mixed with probes before being applied to the tissue and left to hybridize overnight at 50°C. The next day, slides were treated with RNase A (20 mg/L, Sigma-Aldrich) at 37°C for 30 min to remove excess probe, and washed twice in 0.2× saline–sodium citrate buffer. Sections were then blocked twice for 45 min with BM blocking solution (Roche Applied Science) before having an anti-digoxigenin alkaline phosphatase-linked antibody applied at a 1:1,250 ratio to slides for 2 h at RT. The antibody was removed and the tissue was blocked three times in a 1% bovine serum albumin (BSA) block solution (1% BSA, 25 mM NaCl, 0.003% Triton X-100, and 100 mM Tris-HCl at pH 7.5), with the final blocking step being overnight at 4°C. After the overnight incubation in the blocking solution, slides were washed in a substrate buffer (25 mM NaCl, 100 mM Tris- HCl pH 9.7, 50 mM MgCl_2_). The alkaline phosphatase (AP) substrate, 5-bromo-4-chloro-3-indolyl-phosphate/nitro blue tetrazolium (BCIP/NBT; Promega), was prepared according to the manufacturer’s specifications before being applied to the sections. Tissues were incubated from 3 h to 6 h with BCIP/NBT until the color signal was fully developed. Signal development was stopped in 1× Tris-EDTA buffer (10 mM Tris-HCl at pH 7, and 1 mM EDTA at pH 8). Slides were then dehydrated and mounted as previously described in the "GUS Staining" section. Slides were imaged on a Leica DMR fitted with SPOT Insight 4.0 Mp Color F-Mount (SPOT Imaging Solutions) using SPOT advanced software (SPOT Imaging Solutions).

### Immunocytochemical Localization

We used a polyclonal anti-IAA antibody raised against free IAA that was cross-linked to BSA at the carboxyl group in Rabbit (Agrisera). To immobilize IAA by covalent binding to proteins, stages 1 to 5–capitulum clusters from *M*. *inodora* and *S*. *vulgaris* were fixed with 3% (v/v) PFA in 4% (w/v) 1-ethyl-3-(3-dimethylaminopropyl) carbodiimide (Sigma-Aldrich) containing 0.1% (v/v) Triton X-100 (Sigma-Aldrich) for 16 h at 4°C (De Diego et al., 2013). Samples were then washed, dehydrated, and embedded in Surgipath Paraplast Plus paraffin (Leica Biosystems) as described in Coen et al. (1990). Sections of 10 µm were fixed onto Superfrost Plus slides (Thermo Fisher Scientific) and processed for anti-IAA immunolocalization as described in Avsian-Kretchmer et al. (2002) with some modifications. Slides were deparaffinized twice in Histo-Clear II ( Scientific Laboratory Supplies) for 10 min and hydrated in an ethanol-water series. They were further incubated in 100 mM phosphate-buffered-saline (PBS) and then treated with 0.1 mg/mL proteinase K (Sigma-Aldrich) in 100 mM PBS for 20 min at RT. Sections were then washed three times for 5 min in 100 mM PBS and incubated in blocking solution containing 5% (w/v) BSA, 100 mm PBS, and 0.1% (v/v) TWEEN 20 for 1 h at RT. Slides were washed in 5% (w/v) BSA, 100 mM PBS to remove the TWEEN 20, and 200 µL of 1:800 (w/v) dilution of anti-IAA polyclonal antibody (4.38 μg/µL; Agrisera) in 0.1% (w/v) BSA and 100 mM PBS solution were applied to each slide, covered with parafilm and incubated in a humidity chamber for 16 h at RT. To remove excess antibody, slides were washed twice with 0.1% BSA, 100 mm PBS, and 0.1% TWEEN 20 for 15 min each at RT, followed by a 15-min wash with 0.1% BSA and 100 mM PBS solution. A quantity of 200 µL of 1:100 (w/v) dilution of anti-rabbit IgG-AP-conjugate (1 mg/L, Promega) in 0.1% (w/v) BSA and 100 mM PBS solution were applied to each slide, which were covered with parafilm and incubated for 4 h in a humidity chamber at RT. One 15-min wash with 0.1% (w/v) BSA, 100 mM PBS, and 0.1% (v/v) TWEEN 20 was followed by an overnight wash at 4°C. Slides were washed twice with 100 mM Tris-HCL pH 9.7, 50 mM MgCl_2_, and 100 mM NaCl solution for 5 min each at RT followed by the application of 300 µL of ready-to-use western Blue AP substrate (Promega) to each slide, which were then incubated for 15 min to 20 min in a humidity chamber in the dark at RT. Purple-blue color development was stopped and slides were mounted as previously described in the "GUS Staining" section. Slides were observed under the Leica DMR microscope and electronic images were acquired using SPOT advanced software (SPOT Imaging Solutions). Image color balancing and cropping was performed using Adobe Photoshop CS6 (Adobe) and figures were executed using Canvas X software (ADC Systems). PIN1, YUCCA1, and LFY immunolocalization experiments were performed as described in Jackson et al. (1994), using polyclonal antibodies against PIN1 (Eurogentec) at a 1:2,000 dilution; YUCCA1 (Abiocode) at 1:1,000; and LFY (Santa Cruz Biotechnology) at a 1:1,000 dilution, and secondary anti-rabbit IgG-AP-conjugate antibody at 1:400 dilution (Sigma-Aldrich).

### MUG

MUG on *DR5*::*GUS S*. *vulgaris* capitula of different developmental stages (stages 1 to 7) was performed as described in Hull and Devic (1995).

### SEM

SEM was performed on *M*. *inodora* capitula at different developmental stages. Tissue was fixed in the fixation solution (4% [w/v] PFA, 0.01% [v/v] DMSO, 1× PBS, 0.001% [v/v] TWEEN 20, and 0.001% [v/v] Triton X-100) overnight at 4°C. The next day, tissues were dehydrated in an ethanol series (30% [v/v], 50% [v/v], 70% [v/v], 85% [v/v], 95% [v/v], 100% [v/v]) for 30 min per step. Dehydrated tissues then underwent critical point drying using a Polaron critical point dryer (Quorum Technologies) and were mounted onto SEM stubs (Agar Scientific) using carbon tape (Agar Scientific). Mounted stubs were sputter-coated with gold for 2 min using a Polaron E5100 sputter-coater (Quorum Technologies). Samples were then imaged on a Quanta 250 FEG (FEI UK) using the secondary detector.

### Phylogenetic Analysis

The maximum likelihood (ML) phylogenetic tree for *MiRAY2* was obtained using amino acid sequences (99 amino acids were used, which included the conserved TCP- and R-domains). Protein alignments were performed with the ClustalX2 program (Thompson et al., 2002) and the ML tree were generated using the RAXML Web site (https://raxml-ng.vital-it.ch; Stamatakis et al., 2008) with 500 bootstrap replicates. The species abbreviations are as follows: *A*. *majus* (*Am*), Arabidopsis (*At*), *G*. *hybrida* (*Gh*), *H*. *annuus* (*Ha*), *Mohavea confertiflora* (*Mc*), and *S*. *vulgaris* (*Sv*).

### Software and Statistical Analysis

Image color balancing and cropping were performed using Adobe Photoshop CS6 (Adobe) and figures were made on Canvas X (ADC Systems). Tables and data analyses were performed on Excel (Microsoft) and graphs were made on GraphPad 6 (GraphPad Prism).

### Accession Numbers

DNA sequences of *MiLFY* (accession no.: KT593920) and *MiRAY2* (accession no.: KT593921) are available in GenBank.

### Supplemental Data

The following supplemental materials are available.

**Supplemental Figure S1.** Capitulum development, auxin, *RAY2*, and *LFY*.
**Supplemental Figure S2.** Immunolocalization of PIN1, YUCCA1, and LFY during *M*. *inodora* capitulum development.
**Supplemental Table S1.** The number of *M*. *inodora* capitula showing conversion phenotypes after exogenous auxin application.
**Supplemental Table S2.** *M*. *inodora* capitulum phenotypes after various auxin treatments.
**Supplemental Table S3.** Local application of IAA on *M*. *inodora* capitula.
**Supplemental Table S4.** Primer sequences.
